# Use of ChatGPT in Urology and its Relevance in Clinical Practice: Comment

**DOI:** 10.1590/S1677-5538.IBJU.2024.0113

**Published:** 2024-05-10

**Authors:** Hinpetch Daungsupawong, Viroj Wiwanitkit

**Affiliations:** 1 Private Academic Consultant Phonhong Lao People’s Democratic Republic Private Academic Consultant, Phonhong, Lao People’s Democratic Republic;; 2 Medical College Saveetha Institute of Medical and Technical Sciences Saveetha University India Tamil Nadu India Medical College, Saveetha Institute of Medical and Technical Sciences Saveetha University India,Tamil Nadu, India

*To the editor*

We would like to discuss “Use of ChatGPT in Urology and its Relevance in Clinical Practice: Is it useful?” (1). Three questions each on “Primary Megaureter (pMU)”, “Enuresis”, and “Vesicoureteral Reflux (VUR)” were answered in the study using ChatGPT 3.5. Three experts combined two distinct answers for every question to test reproducibility before evaluating the answers for correctness and clarity. The findings demonstrated that ChatGPT offered a broad understanding of the subjects, with acceptable answers for VUR and partially accurate definitions for pMU and enuresis.

Nevertheless, there were flaws, like the pMU definition’s omission of crucial information and the recommendation of pointless tests for pMU and enuresis. Furthermore, incorrect treatment advice for enuresis was given. In conclusion, the information provided by ChatGPT was a mix of exact, insufficient, ambiguous, and occasionally misleading. Future work may focus on improving accuracy by fine-tuning the training data, avoiding ambiguity by providing clear answers, and boosting reliability by incorporating expert comments.

All things considered, the investigation demonstrated the advantages and disadvantages of utilizing ChatGPT for medical inquiries. Even if the AI system can provide some useful information, there are still many areas that may be improved. ChatGPT might be improved to offer more trustworthy and accurate information in the future by resolving the found flaws and adding expert input. Norms or standards can be established, and any ethical conundrums can be looked into, to guarantee that AI-generated content is used in an ethical and transparent manner (2).
